# A zone-plate-based two-color spectrometer for indirect X-ray absorption spectroscopy

**DOI:** 10.1107/S1600577519003898

**Published:** 2019-05-24

**Authors:** Florian Döring, Marcel Risch, Benedikt Rösner, Martin Beye, Philipp Busse, Katharina Kubiček, Leif Glaser, Piter S. Miedema, Jakob Soltau, Dirk Raiser, Vitaliy A. Guzenko, Lukas Szabadics, Leif Kochanneck, Max Baumung, Jens Buck, Christian Jooss, Simone Techert, Christian David

**Affiliations:** a Laboratory for Micro- and Nanotechnology, Paul Scherrer Institut, 5232 Villigen PSI, Switzerland; b Institute of Materials Physics, Georg-August-Universität Göttingen, 37077 Göttingen, Germany; c Photon Science, Deutsches Elektronen-Synchrotron DESY, 22607 Hamburg, Germany; d Max Planck Institute for Biophysical Chemistry, 37077 Göttingen, Germany; e Institute of X-ray Physics, Georg-August-Universität Göttingen, 37077 Göttingen, Germany

**Keywords:** two-color X-ray absorption spectroscopy, zone plates, soft X-rays

## Abstract

X-ray absorption spectroscopy (XAS) is a powerful element-specific technique that allows the study of structural and chemical properties of matter. Often an indirect method is used to access the X-ray absorption (XA). This work demonstrates a new XAS implementation that is based on off-axis transmission Fresnel zone plates to obtain the XA spectrum of La_0.6_Sr_0.4_MnO_3_ by analysis of three emission lines simultaneously at the detector, namely the O 2*p*–1*s*, Mn 3*s*–2*p* and Mn 3*d*–2*p* transitions. This scheme allows the simultaneous measurement of an integrated total fluorescence yield and the partial fluorescence yields (PFY) of the Mn 3*s*–2*p* and Mn 3*d*–2*p* transitions when scanning the Mn *L*-edge. In addition to this, the reduction in O fluorescence provides another measure for absorption often referred to as the inverse partial fluorescence yield (IPFY). Among these different methods to measure XA, the Mn 3*s* PFY and IPFY deviate the least from the true XA spectra due to the negligible influence of selection rules on the decay channel. Other advantages of this new scheme are the potential to strongly increase the efficiency and throughput compared with similar measurements using conventional gratings and to increase the signal-to-noise of the XA spectra as compared with a photodiode. The ability to record undistorted bulk XA spectra at high flux is crucial for future *in situ* spectroscopy experiments on complex materials.

## Abstract 2

A new implementation for XAS measurements based on zone plates is presented. With a two-color scheme, several fluorescence yields can be probed simultaneously.

## 1. Introduction   

X-ray absorption spectroscopy (XAS) is a widely used spectroscopic technique that has gained increasing interest in the last decades because of its capability to obtain electronic as well as spatial information of many different sample classes. In XAS, a core-level electron is excited to an unoccupied state by the absorption of an X-ray photon (Yano & Yachandra, 2009 ▸). Depending on the proximity of the incident photon energy to the absorption edge of the investigated material, XAS is subdivided into near-edge X-ray absorption fine structure (NEXAFS) and extended X-ray absorption fine structure (EXAFS). The former provides element-specific information about the electronic structure of a material whereas the latter provides insights on the local arrangement of the atoms (Stöhr, 1996 ▸; deGroot & Kotani, 2008 ▸; Wende, 2004 ▸; deGroot, 1994 ▸).

Typically, XAS is measured either directly in transmission or via electron or fluorescence yield channels. Although transmission measurements provide a direct method of obtaining X-ray absorption spectra, this option is often not feasible due to the low transmittance for bulk samples, especially in the soft X-ray regime. As an alternative, measurements of the electron yield (EY) and fluorescence yield (FY) offer an alternative of measuring XAS data indirectly, based on the assumed proportionality of the absorption cross section and the emission of electrons or fluorescence photons (Jaklevic *et al.*, 1977 ▸; Gudat & Kunz, 1972 ▸). When comparing EY with FY measurements, it is apparent that EY is very surface sensitive because of the low escape depth of electrons in matter. FY can be more bulk sensitive depending on the photon energy, but can also be applied with high surface sensitivity by using grazing-incidence geometry. Regarding FY measurements, one can distinguish between collecting all the emitted fluorescence integrated over all energies, which is called total fluorescence yield (TFY), or selecting certain emission energy ranges by measuring the partial fluorescence yield (PFY). For the latter, an energy-sensitive detection scheme must be used. In the case of measuring the Mn *L*-edge of La_0.6_Sr_0.4_MnO_3_ (LSMO), which will be of interest here, conventional PFY is dominated by Mn 3*d* to 2*p* transitions, whereas high-resolution PFY also separates the 3*s* to 2*p* transitions located between the oxygen 2*p* to 1*s* emission and the manganese 3*d* to 2*p* emission. In general though, PFY at the Mn *L*-edge requires enhanced energy resolution because of the relatively small energy separation between the different fluorescence channels. Besides TFY and PFY, the measurement of inverse partial fluorescence yield (IPFY) has gained some attention (Achkar *et al.*, 2011 ▸). In this method, one emission energy is chosen for a detailed spectral analysis, where the fluorescence is non-resonant with respect to the X-ray absorption and stems ideally from another element in the compound. Assuming a constant cross section in this channel across the energy range of interest, the reduction of fluorescence in this channel is a direct measure for the absorption at the element of interest. Therefore, this channel also suffers much less from self-absorption effects, making it possible to gain an undistorted bulk X-ray absorption spectrum.

**Figure 1:**
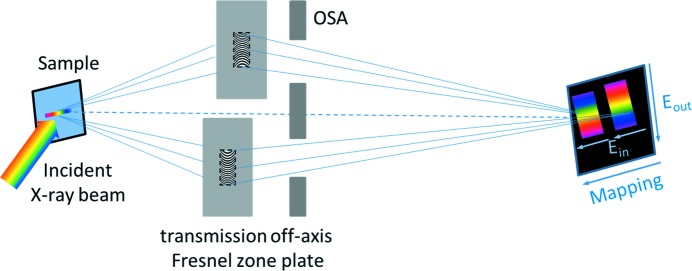
Sketch of the two-color XAS analyzer scheme. Two transmission off-axis Fresnel zone plates are used to collect the emitted X-ray signal from the sample and to disperse it along one axis of the detector, while maintaining spatial resolution along the other axis. This allows for mapping the energy spread of the incident X-ray beam perpendicular to the emitted fluorescence at the same time.

In this publication, we introduce a new analyzer scheme for XAS that uses transmission off-axis Fresnel zone plates (TZPs). These optics are designed in a way that combines energy dispersion in one direction with spatial resolution in the perpendicular direction. The optical setup has already been used for the analysis of resonant inelastic X-ray scattering (RIXS) of solid as well as of liquid samples (Marschall, McNally *et al.*, 2017 ▸; Döring *et al.*, 2018 ▸; Marschall, Yin *et al.*, 2017 ▸). The complete setup consists of two off-axis transmission Fresnel zone plates that collect the emitted fluorescence from the sample and disperse it across a region of the CCD detector (Fig. 1 ▸).

This two-color scheme allows for focusing on two emission energies at the same time so that, under excitation at the Mn *L*-edge, two PFYs (Mn 3*d* to 2*p* transition and Mn 3*s* to 2*p* transition) and the IPFY (O 2*p* to 1*s* transition, resulting from non-resonant O *K*-edge emission) can be detected using both zone plates at the same time. By integrating over the dispersive direction on the detector, even a large part of the TFY can be obtained simultaneously. The possibility to measure in principle both PFYs, IPFY and TFY, with two different zone plates at the same time makes this scheme very efficient and allows high-throughput experiments. In addition, the ability to focus on two different emitted energies can be very interesting for measurements of complex materials or diluted samples, especially for *in situ* measurements where ambient conditions are varied.

## 2. Materials and methods   

**Figure 2:**
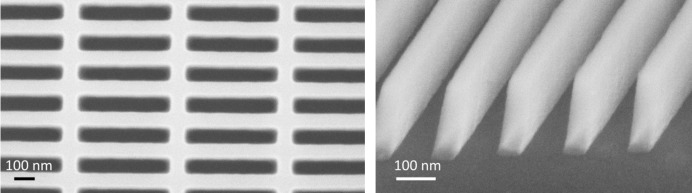
SEM images showing parts of the off-axis zone plates. Left is a top view of the broader inner zone structures. The right image was taken under an angle of 30° showing the height of the zones.

The new XAS analyzer scheme presented here depends strongly on the optical components, namely the off-axis zone plates (see Fig. 2 ▸). They were fabricated by spin-coating a hydrogen silsesquioxane film on a 250 nm-thick Si_3_N_4_ membrane and writing the zone plate structure by high-resolution electron beam lithography (Vistec EBPG 5000Plus). Each off-axis zone plate had a size of 3 mm × 3 mm, an outermost zone width of 35 nm which corresponds to 14285 lines mm^−1^, and a height of around 400 nm.

For the two-color experiments, two different zone plates were necessary for the corresponding energies of the absorption edges of the investigated materials. Their parameters can be found in Table 1 ▸.

The zone plates were placed between sample and detector with the energy-dispersive direction horizontal. In contrast to conventional reflecting variable-line-spacing grating analyzers, zone plates can be mounted very close to the optical axis such that only negligible offset for the beam path has to be considered in the setup. Moreover, zone plates have the advantage of being insensitive to tilt, which allowed us to align the analyzer zone plates within just a few minutes.

Depending on the photon energy and the zone plate period, the zone plates were mounted with a well defined inclination towards the optical axis. This special geometry was chosen in order to benefit from volume effects inside the zone structures and thereby enhancing the efficiency of the analyzer optics (Hambach *et al.*, 2001 ▸). In order to achieve maximum efficiency, we exploited these volume effects and optimized the fabrication process concerning structure aspect ratio, which we pushed towards the current possible limits in nanofabrication (Rösner *et al.*, 2018 ▸). For an estimation of the optimized efficiency, numerical calculations using rigorous coupled wave analysis were performed. In the case of the O zone plate, which was used at around 525 eV, we estimated an efficiency of up to 15%, whereas for the Mn zone plate at 637 eV we calculated an efficiency of up to 14% (Maser & Schmahl, 1992 ▸).

**Figure 3:**
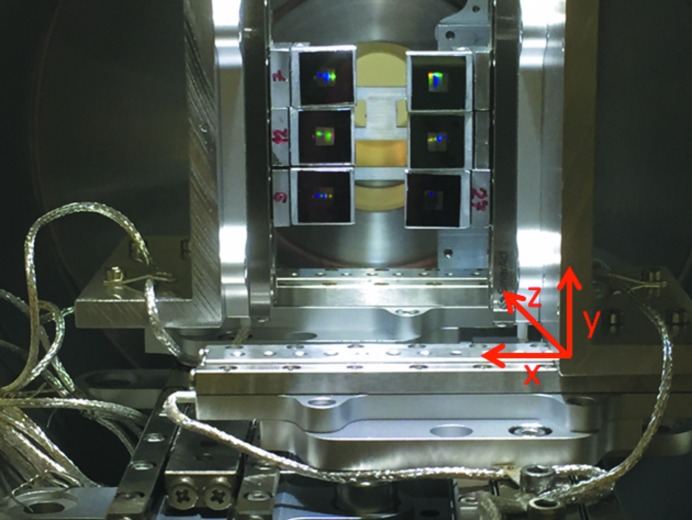
Photograph of the modular optics chamber. Six off-axis zone plates (each specially designed for one particular energy) can be loaded in the chamber. Two of them collect the radiation from the sample and disperse it across the detector. Those can be moved via three axes of translation, each. In the background is an OSA that blocks stray light. The whole setup is mounted on a rotatable axis.

The zone plates, with optimized tilt angles, were mounted on a setup of Smaract nanopositioners, which was implemented in a small vacuum chamber with 153 mm diameter. This modular optics chamber can be easily connected to existing sample chambers. Fig. 3 ▸ shows the inner components of the modular optics chamber, consisting of eight nanopositioners, six off-axis zone plates of which two are used in parallel and an order sorting aperture (OSA). This optics chamber was attached to the ChemRIXS endstation (Yin *et al.*, 2017 ▸), which was installed at the P04 beamline at PETRA III in Hamburg, Germany (Viefhaus *et al.*, 2013 ▸). The ChemRIXS endstation provides a motorized sample stage and allows investigations of liquid jets and for conducting *in situ* experiments of solids in a water vapor atmosphere. The spectrometer arm of this setup is equipped with an ANDOR iKon-M 934 CCD with 1024 pixels × 1024 pixels with a size of 13 µm × 13 µm. The distance between the CCD and sample was 1.33 m and, due to the flexibility of the zone plate chamber, the whole setup could be realized with a straight vacuum pipe without any offsets or bellows. Additionally, two SXUV100 photodiodes (Opto Diode Corp) were mounted to simultaneously record the conventional total fluorescence yield (PD-TFY). The emission energy scale of both zone plates was calibrated by setting the center of gravity of each transition to the tabulated energy of that emission line (*i.e.* O 2*p*–1*s*, Mn 3*d*–2*p*, *etc*.) (Thompson *et al.*, 2009 ▸). The incident energy scale was calibrated by correcting for the shift between the XAS spectrum extracted from the zone plate data (*i.e.* the ROIs giving the TFY, PFY and IPFY) and that of the simultaneously recorded PD-TFY.

Highly crystalline LSMO thin films were chosen to establish the new detection scheme as they are free of pin holes, uniform in structure and composition, and extremely flat. Furthermore, the material is of high interest for fundamental studies of energy storage processes at ambient temperature (Risch *et al.*, 2014 ▸; Scholz *et al.*, 2016 ▸). The LSMO films were prepared by ion-beam sputtering as reported previously (Scholz *et al.*, 2017 ▸). Nb-doped SrTiO_3_ (STNO) 0.5 wt% (CrysTec GmbH) was used as the substrate. The films were deposited at 750°C in an oxygen atmosphere of 1.7 × 10^−4^ mbar. To reduce the amount of preparation-induced defects, the prepared films were kept under preparation conditions for 30 min and slowly cooled to room temperature, including a resting point of 30 min at 500°C. These parameters were selected to yield films of 80 nm thickness as reported previously (Scholz *et al.*, 2016 ▸).

**Figure 4:**
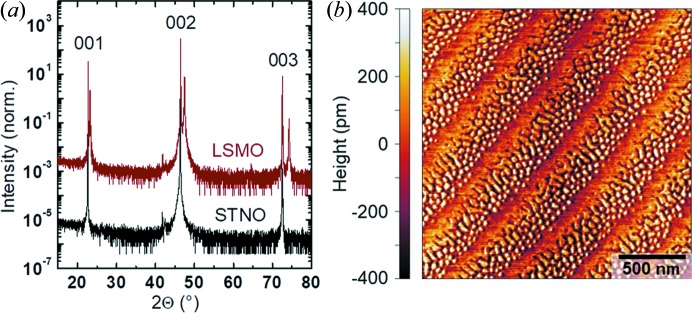
(*a*) Out-of-plane XRD of the used LSMO film (red) and an STNO substrate (black) demonstrating epitaxial growth. (*b*) AFM image of the film demonstrating a very low roughness of the order of a unit-cell step.

Off-axis XRD, as shown in Fig. 4 ▸(*a*), demonstrated that the LSMO grew epitaxially on the STNO substrate, since only the reflections of the {001} family were detected. The LSMO films have the expected double peaks where one matches that of the STNO substrate [*a*_STNO_ = 3.91 Å (Mitchell *et al.*, 2000 ▸)] and the other peak at a higher angle was attributed to the slightly shorter pseudo-cubic lattice parameter of LSMO [*a*_LSMO_ = 3.88 Å (Zemni *et al.*, 2004 ▸)]. Moreover, the films produced were extremely flat as shown by atomic force microscopy (AFM) in Fig. 4 ▸(*b*). The film had a decorated terraced surface that reflects the terrace structure of the STNO substrate. The terraces were about 250 nm wide and the step height is comparable with the pseudo-cubic lattice parameter of LSMO. Overall, the physical properties of the produced films equal those in our previous studies where additional characterization and a discussion can be found elsewhere (Scholz *et al.*, 2016 ▸, 2017 ▸).

## 3. Results and discussion   

**Figure 5:**
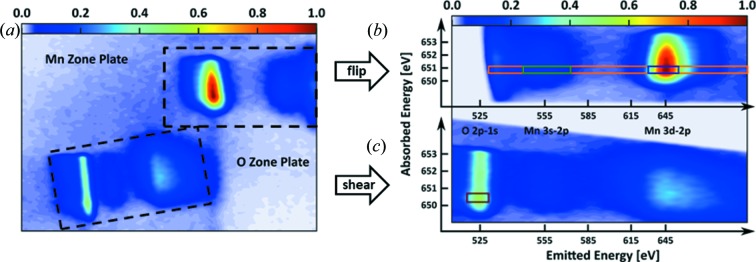
(*a*) Maps of incident and emitted energies from an LSMO sample collected simultaneously using a manganese zone plate (Mn-TZP) and an oxygen zone plate (O-TZP) recorded with the central beamline energy set to 651.5 eV. (*b*) Evaluation of the signal obtained through the Mn zone plate. For easier comparison, the upper spectrum was flipped in order to correspond to the emitted energy axis. (*c*) Contribution of the O zone plate, which was slightly sheared in order to compensate a misalignment that resulted in a lightly tilted setup. Colored boxes correspond to the ROI where the XAS data were obtained as described in the text and evaluated in Fig. 6 ▸. The map was smoothed by Gauss filtering (7σ); the original camera image can be found in Fig. S1 of the supporting information.

We excited an epitaxial LSMO thin film with synchrotron radiation at different soft X-ray energies in the ranges 520–580 eV and 630–665 eV spanning across the absorption edges of O-*K* and Mn-*L*_3,2_, respectively. For maximal flux, the vertical monochromator exit slit was fully opened, which resulted not only in a high number of incident photons but also in a variation of the incident energy along the vertical axis of about 0.5% spread (up to 3.8 eV) over a line of *ca*. 800 µm. An essential advantage of zone plate analyzers is their imaging capabilities that allow for mapping the variable incident energy from the sample to the one axis of the detector and combining this information with the fluorescence information which is perpendicular to it (Marschall, Yin *et al.*, 2017 ▸; Strocov, 2010 ▸). This enabled us to adjust the beamline to one energy value and probe the XAS around this energy by mapping the range of incident energies together with the emitted energies at the same time. Such an acquired signal for one range of incident energies is shown in Fig. 5 ▸.

Fig. 5 ▸ shows a representative detector image, which results from applying the two-color analyzer setup after setting the central beamline energy to 651.5 eV. For each of the imaged transitions, an appropriate coordinate system combining the emitted energy on the *x* axis with the incident photon energy on the *y* axis was overlaid. Opening up the vertical monochromator exit slit increased the photon flux significantly and allowed for measuring incident energies between ∼650 eV and ∼653 eV in a single image. The lack of monochromated excitation is actually advantageous for our new approach as, due to the imaging capabilities of the zone plates, the experimental energy resolution in the direction of the incident energy dispersion is essentially limited by the pixel size, to 14 meV, which is much smaller than the intrinsic lifetime broadening of the core hole (0.4 eV at the Mn *L*_3_-edge and 1.5 eV at the Mn *L*_2_-edge; Fuggle & Inglesfield, 1992 ▸). However, the analysis procedure yielding XAS at this very high resolution is complex and will be discussed elsewhere. Instead, the additional data is used here to reduce the statistical noise of the XAS as discussed below. The possibility to decide in the analysis whether energy resolution of the X-ray absorption spectra or signal-to-noise is of greater importance for that very experiment is one of the unique features of the zone plate analyzer scheme. Together with the high efficiency and the – for zone plate values – large collected solid angle, measurements had an integrated flux of 18.27 × 10^5^ counts s^−1^ under the full Mn-*L*α_1,2_ and *L*β emission lines centered at 642.1 eV (in the range of 3.2 eV incident energy, corresponding to the energy range collected in a single image with the 3*d*-TZP) as compared with 0.91 × 10^5^ counts s^−1^ using a conventional grating and monochromated radiation (Figs. S3 and S4). The flux at the detector was thus up to a factor of 17 higher using our new approach (Table S1).

Two independent maps of incident and emitted energy as imaged by two different zone plates can be collected at the detector at the same time [see Fig. 5 ▸(*a*)]. The image in Fig. 5 ▸(*b*) was focused on the Mn 3*d*–2*p* transition, which sharply imaged the corresponding emission line. To the right, the defocused Mn 3*s*–2*p* transition is also visible before the imaging area ends. In Fig. 5 ▸(*c*), the zone plate was focused on the O 2*p*–1*s* transition. Additionally, the Mn 3*s*–2*p* as well as the Mn 3*d*–2*p* transitions were imaged but defocused. Comparing Figs. 5 ▸(*b*) and 5(*c*), it can clearly be seen that the maximum of the defocused image is lower and it is much wider on the emission axis. Finally, Fig. 5 ▸ illustrates that the images of the emission lines must be separated vertically and horizontally to avoid interference between the signals originating from different zone plates, particularly of the defocused parts.

**Figure 6:**
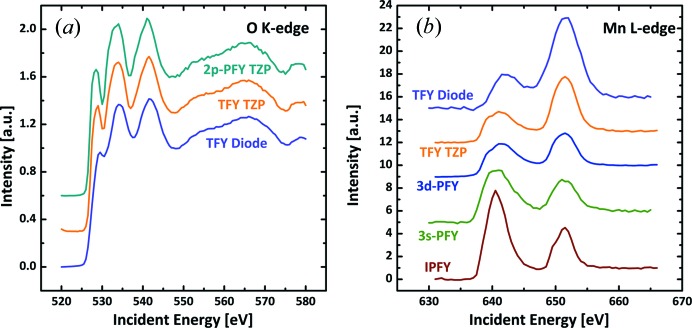
(*a*) Indirect XAS data for excitation at the O *K*-edge and (*b*) at the Mn *L*_3,2_-edges obtained by analysis of the detector images employing the zone plate scheme and a conventional photodiode (violet). All spectra were vertically shifted for clarity.

X-ray absorption spectra were obtained by summing up the counts within a region of interest (ROI) in the obtained camera images measured at beamline energies between 520 eV and 580 eV for the O *K*-edge and 630 eV to 665 eV for the Mn *L*_3,2_-edge, both in increments of 0.5 eV steps. The ROIs were chosen for a width of 0.5 eV around the corresponding incident energy and the FWHM along the emitted energy. The sum in the ROI is then plotted against the beamline energy producing the XAS spectra in Fig. 6 ▸, where they are compared with the output of a photodiode. The signal of the photodiode was also used to align the FY spectra along the incident energy by matching the maxima of the *L*_2_-edges. Note that the color of the ROIs in Fig. 5 ▸ and the color of the spectra in Fig. 6 ▸(*b*) match, yet Fig. 5 ▸ represents only one point in the indirect XAS data excited at the Mn *L*-edge. In the lower energy range, only the core holes in oxygen can be excited, over which the O *K*-edge was scanned [Fig. 6 ▸(*a*)]. This has two consequences, namely that the image of the Mn 3*d* zone plate was not analyzed for this edge and that the spectra obtained by the TFY and PFY are nearly identical to that of the photodiode. On closer inspection, the pre-peak at ∼530 eV was reduced in the spectra of the photodiode, which might be avoidable by an optimized placement of the diode in the measurement chamber. The XAS spectra at the Mn *L*_3,2_-edge differ significantly [Fig. 6 ▸(*b*)]. For these spectra, we used the images of both zone plates, where the Mn 3*d*-PFY, the Mn 3*s*-PFY and the zone plate TFY was obtained on the Mn 3*d* zone plate while the IPFY was obtained from the O 2*p* zone plate. The Mn 3*s*-PFY can also be obtained using the O 2*p* zone plate, which gives a qualitatively similar spectrum (Fig. S2). Here, we used the spectrum from the Mn 3*d* zone plate as it is more in focus. In the extracted XAS spectra, the signal of the Mn *L*_3_-edge was drastically reduced as compared with the Mn *L*_2_-edge for the TFY, either accessed through the zone plates (TFY-TZP), the photodiode (TFY-PD) or the Mn 3*d*-PFY due to self-absorption effects (Eisebitt *et al.*, 1993 ▸). Only the Mn 3*s*-PFY and IPFY spectra showed a qualitatively undistorted spectrum where the Mn *L*_3_-edge is larger than the Mn *L*_2_-edge. A comprehensive discussion and quantification of the distortions are beyond the scope of this manuscript. Nonetheless, we can conclude that our new detection scheme can successfully measure undistorted Mn-*L*_3,2_ XAS in contrast to the conventional photodiode.

Another way of comparing the zone plate analyser with the photodiode is concerning their respective signal-to-noise ratio, as shown in Table 2 ▸. For the calculation of these values, the signal was globally set to the edge jump height, which was normalized to 1. The edge jump (before normalization) is proportional to the total absorption of the material, which gives us the signal. The noise values were calculated as the root-mean-square in a relevant area below the onset of the resonant absorption, namely from 521.5 eV to 524.5 eV for the O *K*-edge and from 632.5 eV to 636.0 eV for the Mn *L*-edge. It is apparent that the signal-to-noise ratio for the zone plate analyzers are about twice that of the classical photodiode when the TFY is compared and even higher for most of the partial yields.

Finally, we would like to point out that this scheme could, in future, also be used for investigations on later transition metals regarding, for instance, the M 3*s*–2*p* emissions (*e.g.* Fe 3*s*–2*p* at 615.2 eV) or the M 3*d*–2*p* transitions (*e.g.* Fe 3*d*–2*p* at 705.0 eV). With the possibility to store more than two zone plates in the optics chamber, these transitions could be captured by changing to a specially made zone plate. Thus, we recommend the two-color scheme with one zone plate focused on oxygen (O 2*p*–1*s* 524.5 eV) for IPFY and a second zone plate focused on the higher transition for PFY.

## 4. Conclusions   

In this work, we have shown the first implementation of off-axis transmission Fresnel zone plates for XAS measurements in a two-color scheme. This scheme offers the advantages of measuring the 3*d*-PFY, 3*s*-PFY, the IPFY and an integrated fluorescence yield resembling the TFY at the same time and with high efficiency. Thus, it allows undistorted XAS data of complex materials with high throughput to be obtained.

## Supplementary Material

Supporting figures and tables. DOI: 10.1107/S1600577519003898/ve5095sup1.pdf

## Figures and Tables

**Table 1:** Parameters of the zone plates used at different energies (Bearden, 1967 ▸; Krause & Oliver, 1979 ▸)

Zone plate ID	O-TZP	Mn-TZP
Transition	O 2*p*–1*s*	Mn 3*d*–2*p*
Photon energy (eV)	524.9	637.4
Object distance (m)	0.18	0.24
Focal length (m)	0.16	0.20
Image distance (m)	1.15	1.09
Outermost zone width (nm)	35	35
Outer radius (mm)	5.24	5.46
Size (mm × mm)	3 × 3	3 × 3

**Table 2:** Signal-to-noise ratios (S/N) for all the spectra recorded with the TZPs in comparison with those of a photodiode (Fig. 6 ▸)

Detection method	S/N
O *K*-edge	
TFY TZP	657
2*p*-PFY	611
TFY PD	354
	
Mn *L*-edge	
TFY TZP	48
3*d*-PFY	46
IPFY	37
3*s*-PFY	22
TFY PD	17
