# Supplementary material for: A zone-plate-based two-color spectrometer for indirect X-ray absorption spectroscopy
Source: J Synchrotron Radiat. 2019 May 24;26(Pt 4):1266–71. doi: 10.1107/S1600577519003898 (PMC6613121; doi:10.1107/S1600577519003898)
Supplement: Supplementary file 1 [file s-26-01266-sup1.pdf]

JOURNAL OF SYNCHROTRON RADIATION

Volume 26 (2019)

Supporting information for article:

A zone plate based two-color spectrometer for indirect X-ray absorption spectroscopy

Florian Döring, Marcel Risch, Benedikt Rösner, Martin Beye, Philipp Busse, Katharina Kubiček, Leif Glaser, Piter S. Miedema, Jakob Soltau, Dirk Raiser, Vitaliy A. Guzenko, Lukas Szabadics, Leif Kochanneck, Max Baumung, Jens Buck, Christian Jooss, Simone Techert and Christian David

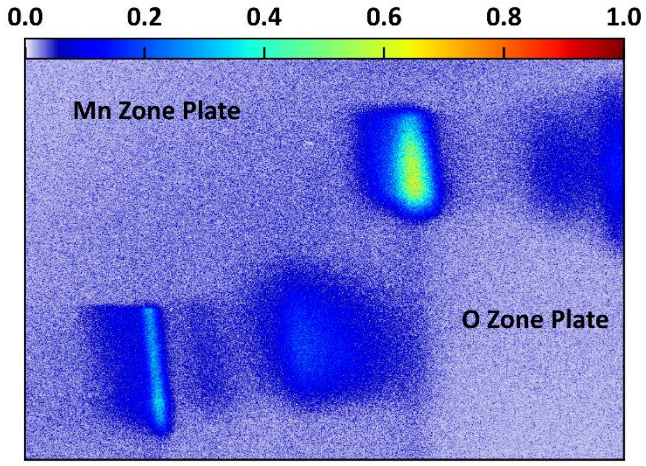

Figure S1 Maps of incident and emitted energies from an LSMO sample collected simultaneously using (a) an Mn zone plate (Mn-TZP) and (b) an oxygen zone plate (O-TZP) recorded with the beamline set to 6.51 eV.

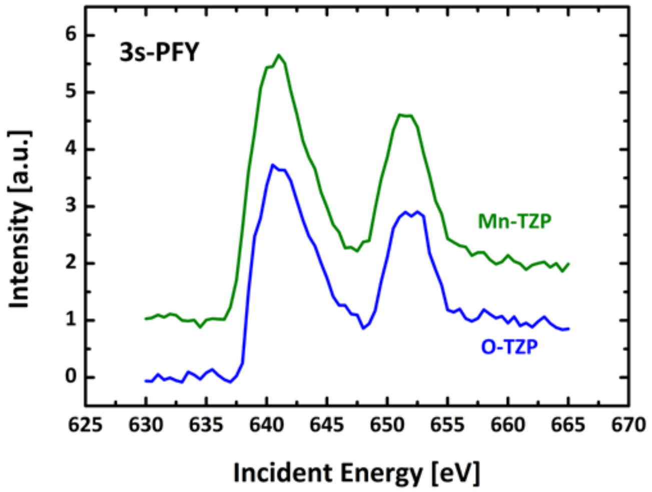

Figure S2 Comparison of the two 3s-PFY spectra achieved via the Mn-TZP (green) and the O-TZP (blue). Both data sets were recorded simultaneously during the same run. It can be seen that the signal to noise ratio is for the Mn-TZP much better than for the O-TZP, even though the focusing of the O-TZP (524.9 eV) is much closer to the Mn 3s-2p transition (556.3 eV) than the focusing of the Mn-TZP (637.4 eV).

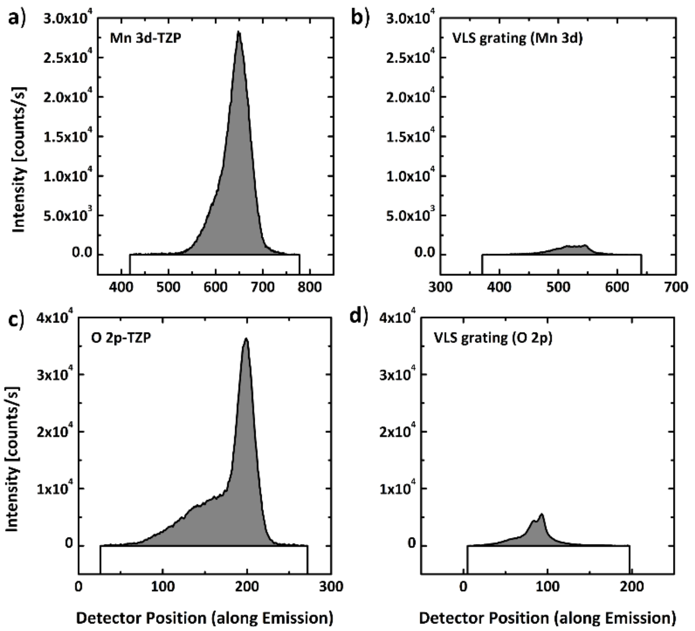

Figure S3 Determination of the efficiency ratios of the TZPs (left) to measurements done with a VLS grating (right). For the same exposure times, the absolute count rates of the relevant transitions (top: Mn 3d-2p, bottom: O 2p-1s) were compared (grey areas). For the analysis, a width of 3.2 eV in absorption energy was integrated. This shows that the Mn-TZP (a) has a 20 times higher efficiency compared to the VLS grating (b). For the O-TZP it results in a higher efficiency by a factor of 8 comparing c) to (d).

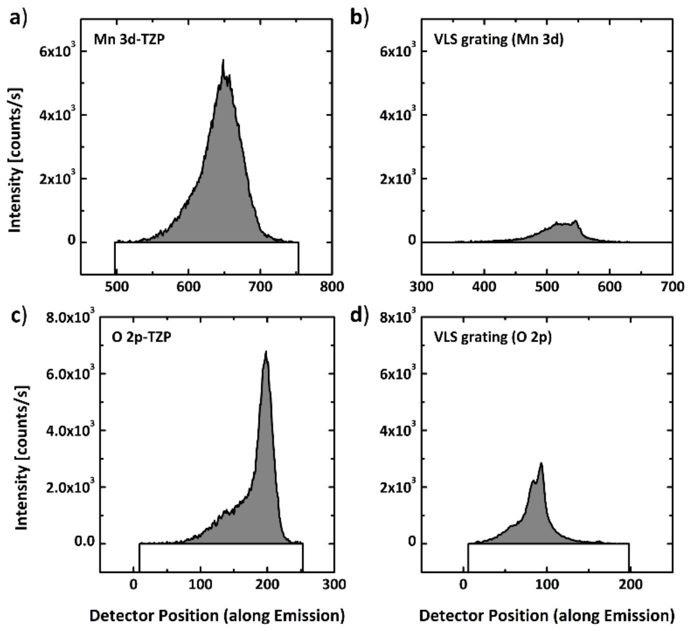

Figure S4 Determination of the efficiency ratios of the TZPs (left) to measurements done with a VLS grating (right). For the same exposure times, the absolute count rates of the relevant transitions (top: Mn 3d-2p, bottom: O 2p-1s) were compared (grey areas). For the analysis, a width of 0.5 eV in

absorption energy was integrated. This shows that the Mn-TZP (a) has a 7 times higher efficiency compared to the VLS grating (b). The same procedure leads for the O-TZP (c) to a higher efficiency by a factor of 3 compared to (d).

Table S1 Comparison of the counts/s for the TZPs and a VLS grating for both regions of absorption energy shown in the figures S3 and S4 resulting in the shown efficiency coefficient. The count rates were obtained by integrating over the full emission of the corresponding transitions shown as grey areas in Figs. S3 and S4. The efficiency coefficient was calculated as the ratio of count rates between the TZPs and the VLS grating.

**Range of absorption energy**	**3.2 eV**	**0.5 eV**
**Counts (Mn 3d-TZP)**	$18.3*10^5$	$3.4*10^5$
**Counts (VLS grating: Mn 3d)**	$0.9*10^5$	$0.5*10^5$
Efficiency coefficient	**20.0**	**6.7**
**Counts (O 2p-TZP)**	$15.1*10^5$	$2.6*10^5$
**Counts (VLS grating: O 2p)**	$1.8*10^5$	$0.9*10^5$
Efficiency coefficient	**8.4**	**2.9**

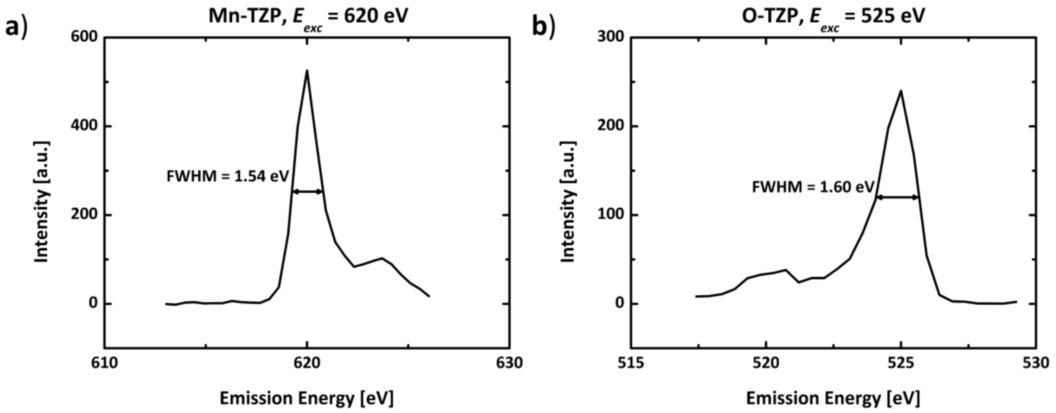

Figure S5 Determination of the resolving power of the emission of the Mn-TZP (a) and the O-TZP (b) using the FWHM of the elastic line scattered by carbon. The ratio E/ΔE of the excitation energy over the energy resolution gives, after removing influences coming from the beamline (Yin et al., 2017), resolving powers of 430 and 350, respectively. These values are mainly limited by the spot size on the sample, and fully sufficient for the application in X-ray absorption spectroscopy.
